# MALVAC 2012 scientific forum: accelerating development of second-generation malaria vaccines

**DOI:** 10.1186/1475-2875-11-372

**Published:** 2012-11-09

**Authors:** Kirsten S Vannice, Graham V Brown, Bartholomew D Akanmori, Vasee S Moorthy

**Affiliations:** 1 Johns Hopkins Bloomberg School of Public Health, Johns Hopkins University, Baltimore, Maryland, USA; 2 Nossal Institute for Global Health, University of Melbourne, Carlton, Victoria, 3010, Australia; 3 Immunization and Vaccines Development Programme, Division for Prevention and Control of Communicable Diseases, WHO Regional Office for Africa, Brazzaville, Congo; 4 Initiative for Vaccine Research, Department of Immunization, Vaccines & Biologicals, World Health Organization, Avenue Appia 20, Geneva, 1211-CH 27, Switzerland

**Keywords:** Global, Malaria, WHO, Malaria vaccine, Elimination, Vaccine research, Surveillance, Clinical trials, Field trials

## Abstract

The World Health Organization (WHO) convened a malaria vaccines committee (MALVAC) scientific forum from 20 to 21 February 2012 in Geneva, Switzerland, to review the global malaria vaccine portfolio, to gain consensus on approaches to accelerate second-generation malaria vaccine development, and to discuss the need to update the vision and strategic goal of the Malaria Vaccine Technology Roadmap. This article summarizes the forum, which included reviews of leading *Plasmodium falciparum* vaccine candidates for pre-erythrocytic vaccines, blood-stage vaccines, and transmission-blocking vaccines. Other major topics included vaccine candidates against *Plasmodium vivax*, clinical trial site capacity development in Africa, trial design considerations for a second-generation malaria vaccine, adjuvant selection, and regulatory oversight functions including vaccine licensure.

## Background

In 2006, the Malaria Vaccine Technology Roadmap [1] was developed following several meetings in North America, Europe and Africa and an extensive consultative process involving input from over 200 scientists. This included the strategic goal to “develop and license a malaria vaccine that has a protective efficacy of more than 80% against clinical disease and lasts longer than four years” by 2025. The Roadmap included 11 agreed priority areas of research, vaccine development, key capacities, and policy and commercialization.

Progress has been made in several of the Roadmap priority areas. The RTS,S/AS01 vaccine is in a Phase III trial; many advances have been made in clinical trial and immunoassay standardization; new antigens are in the development pipeline, and increased efforts have been made to share information with the research and public health communities. Further advances have occurred in formulation process development, strengthening good clinical practice (GCP) field trial capacity, and considerations of clinical development pathways for multiple classes of malaria vaccines. Special attention has been paid to ethics and regulatory pathways, including an efficient and transparent policy process at the World Health Organization (WHO). This process has articulated the 2015 timings for a first WHO malaria vaccine recommendation on use, related to the RTS,S candidate malaria vaccine [2]. Capacity has also been significantly strengthened in ethics and regulatory oversight function in support of clinical evaluation of candidate malaria vaccines, especially in Africa.

Other changes have also occurred since 2006. A gratifying reduction in morbidity and mortality of malaria has been observed in response to scaled-up control measures in many places, with subsequent alteration in population disease susceptibility and immunity leading to altered patterns of disease. The call for eradication has increased efforts to consider impacts of interventions on transmission in addition to morbidity. Another recent landmark was the Malaria Eradication Research Agenda (malERA) initiative, resulting in the publication of a collection of reviews highlighting key research needs for malaria elimination, including malaria vaccine development and a revised, preliminary target product profile (TPP) for a vaccine focused on blocking transmission [3].

The overall objective of this scientific forum convened by WHO was to provide an update on the current global portfolio and to agree approaches to accelerate second-generation malaria vaccine development (for participant list, see the Additional file 1 “List of participants at the MALVAC Meeting: 20–21 February 2012”). The specific objectives included understanding lessons learned from completed malaria vaccine projects, summarizing current rate-limiting steps for second-generation malaria vaccine development, and considering innovative ways to shorten timelines for second-generation development. Special attention was paid to assumptions or data that have changed since the Roadmap consultation process occurred.

### Pre-erythrocytic malaria vaccine portfolios

Research and development of pre-erythrocytic vaccines has progressed furthest, and a number of approaches in the pipeline could contribute to improvements in the second-generation. The importance of well-established correlates of protection and the value of partnerships to increase capacity were emphasized. The recent achievement of consensus on standard operating procedures (SOPs) and protocols for design of controlled human malaria infection (CHMI) studies represents major progress according to the priority area of clinical trial standardization [4]. There was a call for better information exchange with other vaccine development communities (e g, HIV and TB) on experiences with delivery systems, adjuvants and various prime-boost regimens so that efforts are not unnecessarily duplicated, although it was acknowledged there are important disease-specific issues [5].

From data generated by the leading pre-erythrocytic vaccine groups, it is clear that it is possible with certain regimens to achieve anti-circumsporozoite IgG, as well as CD8 T cell responses targeting another pre-erythrocytic antigen, TRAP, that are much higher than those seen under natural exposure and both responses appear to associate with protection against challenge infection. Adenovirus-containing heterologous prime-boost regimens for CD8 T cell induction in humans (using chimpanzee adenovirus strains or AdHu5) have demonstrated some success, but concern remains about persisting with the Ad5 vector because of widespread occurrence of pre-existing antibodies in target populations and alternatives are actively being explored. CHMI has been useful for decision-making and the ability to gain a preliminary indication of efficacy in comparatively small CHMI studies is an important advantage for pre-erythrocytic vaccines, though CHMI studies are only appropriate in closely monitored and highly specialized settings.

Discussion focused on the minimally acceptable profile for programmatic feasibility of prime-boost regimens; how challenge trials can further accelerate timelines in the future; whether field trials measuring incident infection can play a larger role; and the concept of testing antigens to failure. Using incident infection as the primary outcome, measured as blood film positivity or PCR positivity, which can detect 20 parasites/mL (about 100-fold more sensitive than the most sensitive microscopy methods), rather than large clinical trials, is one way to reduce trial size and shorten the development timeframe. A key limitation of incident infection studies is evaluation of duration of protection, which is itself a very important efficacy outcome. There is uncertainty as to whether impact on incident infection correlates well with clinical protection in semi-immune individuals, although there was a good correlation in the case of RTS,S. It was noted that in endemic areas, trial designs that include genotyping often add useful information, and this aspect should be addressed whenever possible. Consensus-based techniques for determining multiplicity of infection and genotyping would be helpful and would be essential if these data were to be used for WHO decision-making. The ability to clear infection before a trial begins was discussed and remains appropriate in the specific case of studies of incident infection.

Go/no-go criteria were also discussed. Although pre-erythrocytic vaccines with no sterile protection in challenge trials do not warrant paediatric evaluation, it was noted that challenge trials are generally small and confidence intervals wide, so the efficacy point estimate in any one study may not reflect the true efficacy of the vaccine. Hence sample size calculations and confidence intervals should be considered carefully when making go/no-go decisions for second-generation vaccines based on challenge trials. As more and more combinations are highlighted as being worthy of clinical testing, the lack of a definitive method to prove a vaccine concept prior to challenge trials means that more challenge trials will be needed. Robust pre-clinical and *in vitro* data will have to be used to limit the number of human trials to a practical number. When considering what is failure, it is likely that RTS,S has set the mark for what is minimally expected, and subsequent vaccines are likely to be considered against the performance benchmarks set by RTS,S, certainly in the pre-erythrocytic arena. It was agreed that the status of malaria vaccine development has progressed markedly in the last few years, most clearly with respect to the pivotal Phase III trial of RTS,S underway. This trial was recognized as a major achievement in its own right.

### Blood-stage malaria vaccine portfolio

Experience with blood-stage vaccine development has shown that down-selection of antigens for clinical testing is not a straightforward process. Multiple blood-stage constructs have reported allele-specific protection in secondary or exploratory analyses, though efficacy results from any one construct have not been replicated [6] and an urgent priority is to replicate results for the same constructs or antigens.

There are no strong immunological correlates of protection, but a great deal of clinical trial data with blood-stage vaccines are now available. Growth inhibitory assays (GIA) correlate only marginally with protection, nevertheless it is considered reasonable to use lack of growth inhibition as a no-go criterion for a vaccine project based on an antigen proposed to induce growth inhibiting antibodies. Experience has not indicated that GIA will predict field efficacy, although there is no comparison yet available between a strongly strain-transcending GIA response and field efficacy. A few antigens have had some effect in clinical testing (i e, AMA1, MSP2 with allele-specific protection, and some possible efficacy for MSP3 from a Phase 1 trial requiring confirmation), but some antigens, such as MSP1, have repeatedly failed.

A more systematic process is needed to eliminate vaccine candidates with clear criteria for advancement in the development process. These criteria may include an understanding of the role of animal studies to predict human responses; which immunological responses in naïve subjects are important; correlation of functional assays with parasite growth rates; consideration of the importance of comparative studies; and the role of CHMI studies for blood-stage vaccines.

The issue of how to address genetic polymorphism was highlighted as a priority issue for this class of vaccines. It was suggested that antigens with extensive genetic diversity should not transition into vaccine development pipelines unless constructs inducing cross-reactive immune responses have been produced.

Additional key topics of discussion included how antigens should be tested to failure, the current view of blood-stage vaccine targets for 2025, and specific issues raised by combining pre-erythrocytic and blood-stage constructs. It was noted that with natural infection there are stronger correlations with protection against multiple antigens and weaker correlations for individual antigens; however, the issues of how to disentangle exposure, age and antigen-specific protection remain challenging.

New methods of antigen presentation coupled with new adjuvants have demonstrated the ability of recently developed vaccines to be better than natural exposure in inducing protective immune responses, a feature seen in the past with few vaccines, tetanus toxoid being one example. Presentation as a viral-like particle was a critical success factor for HPV, and RTS,S has induced a degree of pre-erythrocytic immunity in the field that is not otherwise seen. The impact of the latter on semi-immune children was greater than many anticipated, and this would be a beneficial component of a combination vaccine. Caution was expressed in making assumptions about the magnitude of immune responses needed given the low levels of responses to blood-stage antigens that have been seen compared with the extremely high levels of responses to pre-erythrocytic antigens. For elimination, blood-stage vaccines may not be sufficient by themselves, but they could be an important tool in combination with other antigens and have the added advantage of providing disease modification for breakthrough infection in individuals with waning immunity. Some new ligands may have been identified, and some antibody responses have been consistently detected in individuals who were protected. It was also pointed out that blood-stage vaccines should not be ruled out from the broad class of vaccines that may reduce malaria transmission, although such an effect would need to be demonstrated most likely in post-licensure studies.

### Sexual-stage malaria vaccine portfolio and feeding assays

Two presentations highlighted progress with vaccine constructs based on sexual stage antigens. Pfs25 remains the only sexual stage antigen currently under clinical evaluation, with good pre-clinical progress reported for a construct based on Pfs48/45.

The PATH Malaria Vaccine Initiative (MVI) proposed a minimum TPP for a sexual stage transmission blocking vaccine that includes ≥85% efficacy (yet to be fully defined in this context) and a duration of protection of two years [7]. It was noted that the current TPP for transmission blocking vaccines (TBV) is a high bar, but practically speaking, strong tools are needed if the goal is eradication, and it was argued that the TPP should describe a vaccine that can meet that target. Transmission-related epidemiological data gaps will need to be addressed for trial design in this area, hence the establishment of linkages between groups researching transmission measures and support for epidemiological studies are both critical. Only then will the links between assay measurements and community transmission reduction be understood; this is a data gap at the moment. Great progress has been made in recent years with a general acceptance in malaria vaccine circles that the issue of community benefits for TBV is not a major hurdle for clinical or regulatory pathways. A key issue will be establishing confidence in assays that indicate reduction of infectivity, and what type of assay data would lead to acceptance for decision-making. Each assay has important strengths and limitations in this area: the standard membrane feeding assay (SMFA) may not reflect transmission-blocking activity under field conditions. Direct skin feeding (DSF) and direct membrane feeding (DMF) assays are closer approximations to conditions of natural exposure. There was discussion over whether reduction of oocyst counts was an important and relevant measure of vaccine efficacy, or whether prevention of mosquito infection was the better and more robust endpoint.

Progress has also been made to assess and reduce variability in SMFA, including reagents such as the mosquito species, parasite strain, and monoclonal antibody. Very good progress towards qualification of SMFA was reported at the meeting, and this is a major development for transmission blocking vaccines. The difference between parasites and vectors in laboratory and field settings was raised; techniques need to be properly developed and standardized for African settings.

Key discussion topics included how to advance further with qualification of a membrane-feeding assay for acceptance by decision-makers. It was agreed that this would advance timelines for transmission blocking vaccines. Agreement of a formal trial design for proof-of-concept prior to large-scale cluster randomized trials would be very helpful. Regulatory authorities stated that there was no requirement *per se* for a vaccine to provide individual benefit and there are multiple precedents. Examples of this concept include vaccinations of boys with rubella vaccine to reduce incidence of congenital rubella syndrome, and administration of primaquine to *Plasmodium falciparum* cases, conferring no direct benefit, to reduce transmission. A third example is the requirement to receive a vaccine on entry into a non-endemic country with no direct individual benefit but for prevention of transmission of a disease into the non-endemic area. There was general agreement that, given the substantial public health benefits of a future highly efficacious TBV and the multiple precedents, the lack of direct benefit for an individual should not be an impediment to its development. This consensus in the scientific community should be explored further with regulators as appropriate.

Given the enormous dose requirements for a TBV to be given in mass campaigns, innovative industry partnerships could be explored in order to develop and produce sufficient quantities at reasonable cost. A parallel between the indirect effects of transmission blocking vaccines was drawn with recent developments of CMV vaccines. A key barrier to licensure was the previous focus on a congenital disease endpoint; consensus in the scientific community allowed regulators to agree that prevention of infection in pregnant women was sufficient for licensure, allowing CMV vaccine development to move forward. It was suggested that creative approaches be used for TBV development, and old paradigms should not be binding. For example, one could apply for accelerated approval based on robust data from a qualified assay of infectivity, and use this as a tool to determine the vaccine’s best application. The licensure could be withdrawn if the confirmatory study did not meet the public health needs required of a transmission blocking vaccine.

### *Plasmodium vivax* vaccine research and development

Guidance on clinical evaluation of pre-erythrocytic and blood-stage *Plasmodium vivax* vaccines was published following the 2007 MALVAC meeting and provides recommendations for study design, efficacy and safety endpoints, and study participants [8]. Many principles developed for *P. falciparum* apply to *P. vivax,* and for TBV, all issues are identical, provided allowance is made for relapse from hypnozoites in *P. vivax*. Unique issues for *P. vivax* pre-erythrocytic and blood-stage vaccines include relapses; the need to clear hypnozoites for pre-erythrocytic vaccine evaluation; interference of *P. falciparum* infections; and a theoretical risk of a shift to increased *P. falciparum* infections. The reservoir of infection for each species may be different, with neither accessible solely through the traditional EPI age group (for example, in areas where *P. falciparum* is declining, morbidity is observed in older age groups).

In the five years that have passed since the 2007 recommendations, new issues for consideration include the changing epidemiological context, elimination as a goal, and the possible upcoming regulatory submission of RTS,S for *P. falciparum*. There is much asymptomatic infection, and as even low prevalence of gametocytaemia can contribute significantly to transmission, molecular detection of malaria infection may be required but needs to be standardized for vaccine trials. For *P. vivax*, pre-erythrocytic vaccines are needed to reduce primary infections and relapses; a therapeutic vaccine for hypnozoites would be extremely useful and transmission-blocking vaccines could counter the high transmissibility of *P. vivax*. The molecular force of infection (_mol_FOI), defined as the number of infections with new species clones over time [9], could be used to distinguish between different infections and relapses, and could perhaps be a new endpoint for *P. vivax* vaccine trials. This requires further discussion.

It was highlighted that *P. vivax* is an important disease of adults, so vaccines must also be tested for use in older populations. The need in many situations for a standardized, sensitive tool that can detect all parasites, no matter how low the concentration, was emphasized several times.

The *P. vivax* challenge model is less well established and studied than the *P. falciparum* counterpart. Presentation of a recent *P. vivax* challenge trial led to discussion on whether trial participants could be screened to reduce the risk of relapse. Selection of low-relapsing challenge isolates, optimal screening of volunteers and optimal radical cure regimens will be central to any progress that can be made with *P. vivax* efficacy trials.

Lack of a long-term culture system was emphasized as a critical bottleneck for *P. vivax* vaccine development. The ability to maintain a clonal challenge isolate for *P. vivax* would help reduce some of the complexities inherent in naturally derived *P. vivax* parasite populations. Vaccines that target the hypnozoites are of interest, although they are challenging to test given that relapses can occur even years following infection. One approach could be to use the Chesson strain, which is known to relapse earlier, but doing so would not reflect the real world complexity of the parasite.

More genetic diversity in transmission blocking targets has been seen in *P. vivax* than *P. falciparum*, but whether immune pressure causing heterogeneity occurs with the *P. vivax* hypnozoites or an earlier point in the life cycle is not known. A theoretical risk was expressed (as has been in the past for *P. falciparum*) that a blood-stage vaccine could alleviate symptoms of *P. vivax* without clearing the parasite. In theory this could facilitate development of severe chronic anaemia, as is seen in children of endemic areas, at the same time allowing ongoing transmission. Other malaria species were noted to account for a significant burden of disease in some settings, including *Plasmodium knowlesi, Plasmodium malariae* and *Plasmodium ovale.*

MALVAC was encouraged to continue making *P. vivax* vaccine development a priority as the burden of *P. vivax* is increasing, and conventional methods for control, such as drugs or insecticide-treated bed nets directed against indoor resting mosquitoes, are not effective against outdoor vectors of *P. vivax* and no safe and effective drug is available for hypnozoites. It was suggested that another meeting would be useful to address different trial designs in different geographical regions and how to demonstrate the potential for elimination. Simultaneous consideration of *P. vivax* and *P. falciparum* will be necessary at some point, for they occur together in many areas.

### Sustainable capacity for malaria vaccine trials: current status and five-year view

Experts are needed in endemic countries to keep pace with needs for basic science, clinical trials, and the regulatory demands associated with new products in order to maximize gains and translate results into sustainable public health benefit. Critical resources include a pool of trained professionals, physical infrastructure, capacity for GCP compliance, and functional clinical, laboratory, regulatory, field, and logistic systems. The Malaria Clinical Trials Alliance (MCTA) has helped to create 15 functional Phase II-IV centres (11 of which are participating in the RTS,S Phase III study). MCTA has facilitated government funding for research and strengthened staff capacity but greater capacity is required for Phase I trials, immunological and molecular assays, and data analysis.

Sustaining ongoing regional networks, developing sites in areas currently lacking facilities, ensuring appropriate human resources and retention, and integrating with other drug or vaccine activities are areas that all need further attention. There was much discussion about the need to integrate maintenance costs and on-going surveillance activities into facility budgets. Ownership of trial sites is a key issue and governmental support for trial sites, such as in Tanzania and Mali, was applauded. National ownership is important, and sites should be linked with universities to create teaching opportunities and bridge the gap when trials are not ongoing. The European and Developing Countries Clinical Trials Partnership (EDCTP) has taken this issue seriously and makes capacity building an integrated activity of all trials, including partnerships between established and emerging institutions. Close networks and disease diversification are important for sustainability. Other organizations, such as the European Vaccine Initiative (EVI) facilitate scientific and financial management training, masters programmes, and independent grant awards, and it recognizes achievement of successful future funding as a key output of their investment. The importance of building strong career paths, such as through post-doctoral positions to continue to train scientists and build leaders in the field, is necessary to maintain institutional capacity and memory. Greater capacity in Phase I trials will help sites with continual work flow, since Phase III trials are far more sporadic in nature.

It was suggested that for sustainability, clinical trial sites should be viewed as population health or clinical research centres rather than clinical trial sites that will run down when current trials are completed in order to emphasize their utility across a broad scientific agenda and range of population health challenges, over a long time rather than only for the immediate needs of a particular trial. Most sites discussed already had a demographic surveillance system (DSS), and those that did not are establishing them. Site capacity is also needed in non-African countries for *P. vivax* vaccine studies. In addition to clinical trials, there should be progress in pre-clinical studies and manufacturing of vaccines as well, as this capacity is virtually absent in Africa with the exception of South Africa.

### Strategic views on second-generation malaria vaccine development

Field trial designs for second-generation vaccines will change if a licensed first-generation malaria vaccine becomes available as a comparator. It is also important to convey the need for a second-generation vaccine to ethics committees and national governments. Given decreases in disease incidence, sample size considerations may become challenging in some settings, and vaccine impact on older age children will require assessment. Whether RTS,S is used as a comparator vaccine is an important issue for future studies across all phases. Careful consideration will need to be given to which is the appropriate hypothesis to test at each stage, i e, equivalence, superiority, or non-inferiority. More sites may be needed in areas with higher transmission, particularly as malaria cases decrease and control measures are used at different levels and scaled up at different times and rates. If licensed, RTS,S would be considered for introduction in the context of full implementation of available control methods, and the combined intervention would be expected to help to control malaria, but a second-generation vaccine is likely to be needed to eliminate malaria in much of Africa. It was noted that while continued decreases in malaria burden are highly desirable and achievable, one cannot predict the malaria transmission picture in five to 10 years’ time.

Key strategic issues discussed included rate-limiting steps for second-generation vaccine development, lessons learned, achievements, and the appropriateness of the current target group (infants receiving the routine EPI schedule). WHO recommendations carry much weight, so complexities and considerations need to be carefully specified in relevant WHO documents to allow for flexibility at the country level. It was also noted that going outside the routine EPI schedule for vaccine administration presents a number of barriers, as the same infrastructure for vaccine administration does not currently exist for older age groups in many settings. If mass campaigns are required, increasing the number of doses may affect coverage obtained and impact may be quite different from vaccine efficacy observed in clinical trials.

The importance of communication to explain the likely impact of a partially effective vaccine was emphasized. It should be stressed also that there are precedents for 50% or less efficacy with some other vaccines, such as rotavirus vaccine in some settings, and for some endpoints such as efficacy against pneumonia for pneumococcal conjugate vaccines. Furthermore, if resistance to drugs or insecticides increases, the relative value of any vaccine may increase even with suboptimal efficacy. Any use of a vaccine in women of child-bearing age or pregnant women would require careful consideration and planning for assessment in the women and their offspring, but for ethical reasons delays should be avoided in getting effective vaccines to this susceptible target group.

### Lessons learned for antigen and adjuvant selection, access and formulation capacity

A presentation argued that antigens should be thoroughly investigated and optimized to reduce or preferably even eliminate the need for an adjuvant. A key challenge is assessing how close structurally the antigen is to the form presented in the parasite for induction of protective responses. A clear rationale should exist for the decision to move forward with a novel adjuvant because of the many hurdles associated with its development and licensure. Substantial time and resources are required to develop new adjuvants and data on desired immune responses and how to induce them are emerging only slowly. Early clinical trials are so small that it can be hard to make clear comparisons between different adjuvant candidates or formulations.

A systematic approach to choosing and optimizing an antigen with or without an adjuvant is needed. When there are safety concerns, even if coincidental, it can be hard to exonerate the adjuvant, reinforcing the benefit of using adjuvants that have already been tested in large trials or in licensed products. Microarrays may be a useful tool for understanding which genes are activated during a vaccine-induced immune response and how to develop a long-lasting immune response. Subset analysis of immune responses may assist in definition of epitopes or relevant cytokines responsible for immune induced protection. It was agreed that more research is needed to understand immune mechanisms and their impact on vaccine effectiveness and durability.

### Regulatory considerations for second-generation malaria vaccines

In vaccine licensure submissions to date, vaccine efficacy has been assessed based on direct benefit, and indirect effects have not been primary factors to support licensure. However, it was highlighted that the US regulations do not specifically require vaccines to confer direct clinical benefit on the vaccine recipients. Randomized field efficacy trials are the gold standard for demonstrating efficacy of a vaccine, including second-generation vaccines. Some ethics review boards may consider randomization to a placebo as unethical if there is an approved product available as part of best practice, meaning that comparative studies would have to be larger. There is no precedent for human challenge studies being the primary basis for vaccine approval in the USA, although a US Food and Drug Administration (FDA) expert advisory committee supported challenge trials in concept for cholera vaccines for travellers, which would only need to provide short-term protection [10]. Challenge studies are limited in that they evaluate short-term protection only and could not be done in children, one of the target age groups for a malaria vaccine. The animal rule [11], a US regulation that outlines evidence needed to demonstrate product efficacy when human studies are not possible, is not applicable to malaria vaccines, since human efficacy studies are both ethical and feasible. A surrogate endpoint (such as incident parasitaemia) could be used to support effectiveness of a vaccine if there is scientific consensus that it accurately predicts the true outcome of interest. Accelerated approval (21 CFR 601 Subpart E for US regulations) based on a surrogate endpoint that is reasonably likely to predict clinical benefit is possible for products that would provide a meaningful therapeutic benefit over existing treatments, but it is subject to a required confirmatory study if there is uncertainty regarding a surrogate endpoint. Immune responses are sometimes used to infer effectiveness of a vaccine when it cannot be measured by cases of disease, such as if the disease incidence is very low. However, there must be scientific consensus that the measured immune response is meaningful, and in the case of pre-erythrocytic malaria vaccines, it could only be used if the antigen were the same as in the first-generation vaccine and immunological assays of a known immunological correlate are sufficiently standardized and validated. Superiority of one vaccine over another usually cannot be determined based on immunological responses alone, and, in general, FDA approval of superiority claims is rare.

In the discussion it was pointed out that one must distinguish between statistical validity and the meaning of different measures to benefits for public health. WHO’s position is that incidence of all episodes of clinical malaria is an appropriate measure, both statistically and in terms of relevance to public health. It is also clear that the efficacy estimates apply only to specified durations of follow-up. Caution was given to interpreting vaccine efficacies based on different measures, such as risks, hazards, or rates. Several non-malaria vaccines have been licensed using efficacy measures based on hazards or rate ratios, such as in the field of rotavirus vaccines. It was pointed out that non-inferiority trials are designed to show one product is not unacceptably worse than another (rather than as good as, see Table 1 for field trial design options). The size of the non-inferiority margin needs justification; 5% and 10% were stated as margins that have been previously accepted for products for other diseases (see Table 2 for indicative sample size calculations). There are no regulations specifying how safe and effective a product must be to be licensed, and public health considerations, such as the severity of the outcome the product prevents or treats and the number protected, are considered in an assessment of the overall risks and benefits. Superiority tests will require larger sample sizes, as do small margins for demonstration of non–inferiority. Duration of protection will be a key factor for second-generation vaccines in addition to the magnitude of initial efficacy.

**Table 1:** Considerations of different field trial design options for second-generation malaria vaccines

**Field efficacy trial options**	**2**^**nd**^**generation *vs *placebo**	**2**^**nd**^**generation *vs *1**^**st**^**generation**	**1**^**st**^**/2**^**nd**^**generation *vs *1**^**st**^**generation**	**1**^**st**^**/2**^**nd**^**generation *vs *1**^**st**^**generation *vs *placebo**
Estimate of efficacy	Absolute efficacy estimated.	Relative efficacy estimated.	Relative efficacy estimated.	Absolute and relative efficacy estimated.
Type of assessment	Superiority to no treatment.	Non-inferiority to 1^st^ generation or superiority to 1^st^ generation.	Superiority to 1^st^ generation.	Superiority to 1^st^ generation and to no treatment.
Limitations and Considerations	May be considered unethical to randomize to placebo, if 1^st^ generation vaccine is available and recommended.	Large sample sizes may be needed. Non-inferiority design would not show progress towards the 80% effective goal in the label, but could make alternative vaccines available.	Large sample sizes may be needed. 1^st^ and 2^nd^ generation vaccines could be given together or as prime-boost strategy.	Very large sample sizes may be needed (may not be feasible). May be considered unethical to randomize to placebo, if 1st generation vaccine is available and recommended. This design would not demonstrate efficacy of the 2^nd^ generation vaccine independent of the 1^st^ generation vaccine.
	Efficacy relative to 1st generation vaccine would not be known	Efficacy relative to no treatment would not be known.	Efficacy relative to no treatment would not be known.	

**Table 2:** Examples of total sample sizes required for field studies of second-generation vaccine to demonstrate non-inferiority or superiority (reported in 1,000s; assuming intention-to-treat)

**Power (1-β)**		**: 80%**	
Two-sided significance level (α)		: 5%	
Control vaccine efficacy		: 50%	
Follow-up time		: 2 years	
Incidence rate in those not vaccinated		: 1/10 PYs	
**New vaccine efficacy (%)**	**Superiority**	**Non-inferiority margin**	
		**5%**	**10%**
50	*---*	*28.7*	*7.3*
55	27.4	*7.0*	*3.2*
60	6.6	*3.0*	*1.7*
65	2.9	*1.6*	*1.1*

Sample sizes are reported in 000’s and calculated using Z-test with continuity correction.

### Discussion and recommendations on key issues

The meeting was a timely review of the landscape for new information relevant to second-generation malaria vaccine development and changes that may impact priorities, target age groups, and study designs. The group felt that WHO can make a unique contribution to global efforts to shorten the timeline to the goal of a vaccine that is at least 80% effective against malaria. As independent and open bodies, WHO and MALVAC have important roles in gap identification, convening, consensus building, developing guidance, and facilitating joint planning, with the goal of speeding up the development of a second-generation vaccine.

The following key discussion points were highlighted:

•It is now timely to revisit the vision and 2025 goal highlighted in the Malaria Vaccine Technology Roadmap, considering the changing epidemiology in some places.

•Malaria vaccines will not be used in isolation but rather must be considered in the context of other prevention measures, rapid diagnostic testing and effective treatments and in areas where elimination is being achieved. There may be different scenarios where a malaria vaccine should or should not be used.

•Some earlier assumptions, such as distribution through routine infant programmes, need reconsideration as a result of the changing malaria epidemiology in some places where increasing age of first episode of disease or low transmission could broaden the vulnerable group and hence the target age group for vaccination (again in the 2025 timeframe and beyond).

•*Plasmodium vivax* should not be forgotten as it is a major contributing factor to malaria burden as a whole. It presents unique and complicated issues with the occurrence of relapse and more basic scientific research is needed.

•Success with RTS,S should not slow research towards a second-generation vaccine to meet the desired 80% efficacy goal. Despite challenges due to parasite diversity, many new vaccine candidates look promising.

•There are still urgent unanswered questions concerning optimal malaria assay methods, optimal animal models and the association between assay readouts and real-world experiences.

•Assays for transmission-blocking activity could help rationalize field trials of vaccine candidates, but much work needs to be done on standardization in this area. Major progress towards qualification of one of these assays (the SMFA) was presented for the first time at this meeting.

•Modelling can be useful in understanding the impact of new vaccines, but it must be done in the context of other interventions and with an understanding of key parameters driving uncertainties and the limitations in available epidemiological and immunological data to guide model parameterization.

•There do not appear to be major regulatory hurdles to TBV from the US perspective.

•In the long-term, TBV and pre-erythrocytic vaccines may be combined with blood-stage vaccines in order to prevent severe disease or death from breakthrough infections in subjects with little or waning naturally acquired clinical immunity.

•The need for increased basic research capacity, trial capacity and regulatory authority capacity in Africa is a clear requirement for sustainability. This relates to the entire vaccine development process, from physical infrastructure for basic science and early clinical trials all the way through to regulatory authorities and post-licensure safety surveillance. The creation of career paths to retain talent and enhance leadership is critical.

•Partnerships between the public and private sectors have been shown to be critical for advancing malaria vaccine development, and should be further strengthened.

MALVAC identified a number of areas that would benefit from further WHO involvement:

•The Malaria Vaccine Technology Roadmap, particularly the 2025 targets from 2006 should be reviewed and updated in light of the changing epidemiology. This should be done with a light touch and would include revisiting the target groups.

•Further subpopulations should be considered, such as migrants, transient populations, pregnant women, immunocompromised people, and others, with the needs of the consumer at the forefront.

•The role for TBV and gaps in their development needs to be defined. There are precedents for vaccines judged primarily on indirect effects.

•There is a role for a standardized assay for mosquito infectivity that should be moved into the field when ready.

•Replication of blood-stage vaccine efficacy results is a priority for decision-making.

•Capacity building is needed across the spectrum of vaccine development, from basic research through to regulatory approval.

•The need to maintain momentum around *P. vivax* was clear, particularly as it will be a key challenge for elimination. The best format through which to continue the work was not clear, but could include another meeting or consultations. Consideration of *P. vivax* should be linked around epidemiology, biology, and elimination.

•It is also necessary to keep the other species, such as *P. ovale* and *P. malariae*, in mind.

•Finally, additional consideration needs to be given to future malaria vaccine field studies in the presence of a licensed first-generation malaria vaccine. This should be revisited at the time of policy recommendations for RTS,S in 2015.

## Abbreviations

WHO: World health organization; MALVAC: Malaria vaccine advisory committee; GCP: Good clinical practice; MalERA: Malaria eradication research agenda; TPP: Target product profile; SOP: Standard operating procedure; CHMI: Controlled human malaria infection; HIV: Human immunodeficiency virus; TB: Tuberculosis; PCR: Polymerase chain reaction; GIA: Growth inhibitory assays; HPV: Human papillomavirus; MVI: Malaria vaccine initiative; TBV: Transmission-blocking vaccine; SMFA: Standard membrane feeding assay; DSF: Direct skin feeding; DMF: Direct membrane feeding; CMV: Cytomegalovirus; EPI: Expanded program on immunization; molFOI: Molecular force of infection; MCTA: Malaria clinical trials alliance; EDCTP: European and developing countries clinical trials partnership; EVI: European vaccine initiative; DSS: Demographic surveillance system; FDA: Food and drug administration; CFR: Code of federal regulations.

## Competing interests

All authors declare that they have no competing financial interests.

## Authors’ contributions

All authors were present at the Scientific Forum. KV drafted the initial version of the manuscript. VM coordinated writing of the subsequent drafts of the manuscript. GB, VM and BA edited the second and final draft. All authors read and approved the final manuscript.

## Supplementary Material

Additional file 1 **List of participants at the MALVAC Meeting: 20-21 February 2012.** Click here for file
