# Supplementary material for: MALVAC 2012 scientific forum: accelerating development of second-generation malaria vaccines
Source: Malar J. 2012 Nov 9;11:372. doi: 10.1186/1475-2875-11-372 (PMC3519521; doi:10.1186/1475-2875-11-372)
Supplement: Additional file 1 — List of participants at the MALVAC Meeting: 20-21 February 2012. [file 1475-2875-11-372-S1.docx]

**Additional File: List of participants at the MALVAC Meeting: 20-21 February 2012**

| **Professor Tsiri Agbenyega**, Head of Malaria Research Unit, Department of Child Health, School of Medicines, Kwame Nkrumah University of Science and Technology, Kumasi, Ghana  **Dr Ripley Ballou,** Vice President, Global Vaccines Development, GlaxoSmithKline Biologicals, Rixensart, B - 1330 Belgium  **Dr John W Barnwell*,** Division of Parasitic Diseases, Centers for Disease Control and Prevention, Chamblee, 30341 GA, USA  **Dr Sophie Biernaux,** Head of Malaria Vaccine Development Leadership Team, GlaxoSmithKline Biologicals, B-1330 Rixensart, Belgium  **Professor Fred Binka*,** Dean, School of Public Health, University of Ghana, Legon, Ghana  **Dr Kalifa Bojang***, Malaria Programmme, Medical Research Council Laboratories, Banjul, Gambia  **Professor Graham Brown*, (Chair),** Nossal Institute for Global Health, The University of Melbourne, Parkville, 3050 Victoria, Australia  **Dr Giampietro Corradin**, Institute of Biochemistry, University of Lausanne, Epalinges, 1066 Lausanne, Switzerland  **Dr Hernando del Portillo**, ICREA Research Professor, Barcelona Centre for International Health Research (CRESIB, Hospital Clínic-Universitat de Barcelona), Barcelona, Spain  **Dr Carter Diggs**, Senior Technical Advisor, United States Agency for International Development, Washington, DC 20523, USA  **Dr Carlota Dobaño,** Associate Research Professor, Barcelona Centre for International Health Research (CRESIB, Hospital Clínic-Universitat de Barcelona), Barcelona, Spain  **Professor Ogobara Doumbo,** Professor and Head, Department of Epidemiology of Parasitic Diseases, Malaria Research and Training Center, Bamako, Mali  **Professor Blaise Genton*,** Infectious Disease Service, University Hospital, Lausanne, Switzerland  **Professor Brian Greenwood,** Professor of Tropical Medicine, Department of Infectious and Tropical Diseases, London School of Hygiene and Tropical Medicine, London, WC1E 7HT, UK  **Dr Lee Hall,** Chief, Parasitology & International Programs Branch, Division of Microbiology & Infectious Diseases, NIH/NIAID, Bethesda, 20892 MD, USA  **Professor Adrian Hill,** Jenner Institute, University of Oxford, UK  **Dr Stephen L. Hoffman,** Chairman, Sanaria Inc., 9800 Medical Center Drive, Suite A209, Rockville, MD, 20850, USA  **Professor Toshihiro Horii,** Dept of Molecular Protozoology, Osaka University, Suita, 565 0871 Osaka, Japan  **Dr Babatunde Imoukhuede,** Director, Clinical and Regulatory Affairs, European Vaccine Initiative, D-69120 Heidelberg, Germany  **Dr Robert Johnson*,** Director, Office of Regulatory Affairs, National Institute of Allergy and Infectious Diseases, Bethesda, MD 20892-6604, USA  **Dr David Kaslow,** Director, PATH Malaria Vaccine Initiative, 455 Massachusetts Avenue NW, Suite 1000, Washington, DC 20001, USA  **Dr Didier Leboulleux,** Associate Director Clinical, Malaria Vaccine Initiative (MVI), Program for Appropriate Technology in Health, F-01210 Ferney-Voltaire, France  **Dr Cynthia Lee,** Director, PATH Malaria Vaccine Initiative, 455 Massachusetts Avenue NW, Suite 1000, Washington, DC 20001, USA  **Dr Odile Leroy,** Director, European Vaccine Initiative, D-69120 Heidelberg, Germany  **Professor Mike Levine**, Director, Center for Vaccine Development, University of Maryland School of Medicine, Baltimore 21201-1509, 21201-1509 MD, USA  **Dr Carole Long,** Chief, Malaria Immunology Section, Laboratory of Malaria and Vector Research, 12735 Twinbrook Pkwy, Rockville, MD 20892, USA  **Dr Michael Makanga,** Director South-South Cooperation and Head of Africa Office, European and Developing Countries Clinical Trials Partnership, Tygerberg, 7505 South Africa  **Dr Kamini Mendis,** Independent Consultant, Colombo 5, Sri Lanka  **Professor Paul Milligan*,** London School of Hygiene and Tropical Medicine, London, WC1E 7HT, UK  **Professor Malcolm Molyneux**, University of Malawi, Blantyre, Malawi  **Dr Merribeth Morin,** Program Advisor, PATH Malaria Vaccine Initiative, 455 Massachusetts Avenue NW, Suite 1000, Washington, DC 20001, USA  **Dr Ivo Mueller,** Centre de Recerca en Salut Internacional de Barcelona (CRESIB), Universitat de Barcelona, E-08036 Barcelona, Spain  **Dr Maria Grazia Pau**, Programme Management, Crucell, Leiden, 2301 CA Netherlands  **Dr Inmaculada Penas Jimenez**, European Commission, Brussels 1050, Belgium  **Dr Thomas Richie,** US Military Malaria Vaccine Program, Naval Medical Research Center/Walter Reed Army Institute of Research, Silver Spring, MD 20910-7500, USA  **Professor Robert Sauerwein,** Department of Medical Microbiology/Parasitology (MMB 574), University Medical Center St. Radboud, 6500 HB Nijmegen, Netherlands  **Dr Hanneke Schuitemaker,** Crucell Switzerland AG, 3018 Bern, Switzerland  **Professor Sodiomon Bienvenu Sirima,** Centre National de Recherche et de Formation sur le Paludisme, Ouagadougou 01, Burkina Faso  **Professor Peter Smith,** Department of Infectious Disease Epidemiology (Chair JTEG), London School of Hygiene and Tropical Medicine, London, WC1E 7HT, UK  **Professor Thomas Smith**, Department of Epidemiology & Public Health, Swiss Tropical & Public Health Institute (Swiss TPH), Basel, 4002 Switzerland  **Dr Val Snewin**, International Activities Manager, The Wellcome Trust, London NW1 2BE, UK  **Dr Kenneth Stuart,** Founder, President Emeritus and Member, Seattle Biomedical Research Institute, Seattle, 98109 WA, USA  **Professor Marcel Tanner,** Director, Swiss Tropical & Public Health Institute (Swiss TPH), 4002 Basel, Switzerland  **Professor Mahamadou A. Thera***, Professor of Parasitology-Mycology, Scientific Director, Bandiagara Malaria Project, Faculty of Medicine, University of Bamako, Bamako, 1805 BP, Mali  **Dr Aissatou Toure-Balde*,** Laboratoire d'Immuno-Parasitologie, Institut Pasteur, B.P. 220, Dakar, Senegal  **Ms Kirsten Vannice,** John Hopkins Bloomberg School of Public Health, Baltimore, 21205 Maryland, USA  **Dr Gerald Voss,** Senior Scientist for Emerging Diseases, GlaxoSmithKline Biologicals, Rixensart, B - 1330 Belgium  **Dr Ulrike Wille-Reece,** Program Advisor, Program for Appropriate Technology in Health, Washington, DC 20001, USA  **Dr Janet Wittes**, President, Statistics Collaborative, Inc., Washington DC 20036, USA  **Dr Yimin Wu,** Laboratory of Malaria Immunology and Vaccinology, 5640 Fishers Lane  Bethesda, MD 20892, USA  WHO SECRETARIAT  **Professor Bartholomew Dicky Akanmori**, Technical Officer, Vaccine Regulatory Support, WHO AFRO  **Dr Vasee Moorthy**, Technical Officer, Initiative for Vaccine Research, WHO HQ  **Dr Sergio Nishioka**, Scientist, Quality, Safety and Standards, WHO HQ  **Dr Uli Fruth**, Technical Officer, Initiative for Vaccine Research, WHO HQ  *MALVAC member |
| --- |
